# Therapeutic strategies for uncomplicated cystitis in women

**DOI:** 10.3205/id000086

**Published:** 2024-04-29

**Authors:** Kurt G. Naber, Jakhongir F. Alidjanov, Reinhard Fünfstück, Walter L. Strohmaier, Jennifer Kranz, Tommaso Cai, Adrian Pilatz, Florian M. Wagenlehner

**Affiliations:** 1 Department of Urology, Technical University of Munich, Germany; 2 Biostatistics & Data Science R&D, Bionorica SE, Neumarkt, Germany; 3 Sophien- and Hufeland Hospital, Weimar, Germany; 4 Medical School Regiomed, Coburg, Germany; 5 Julius Maximilian University, Wuerzburg, Germany; 6 University of Split, Croatia; 7 Department of Urology and Pediatric Urology, University Medical Center RWTH Aachen, Germany; 8 Department of Urology and Kidney Transplantation, Martin Luther University, Halle (Saale), Germany; 9 Department of Urology, Santa Chiara Regional Hospital, Trento, Italy; 10 Clinic for Urology, Pediatric Urology and Andrology, Justus Liebig University of Giessen, Germany

**Keywords:** cystitis, urinary tract infection, diagnosis, antibiotic therapy, non-antibiotic therapy, prophylaxis, Acute Cystitis Symptom Score, antibiotic resistance

## Abstract

Uncomplicated cystitis is affecting many women of all ages and has a great impact on the quality of life, especially in women suffering from recurrent, uncomplicated cystitis. By far the most frequent uropathogen, *E. coli*, may have acquired increasing resistance against a variety of oral antibiotics, which may differ between countries and regions. Therefore, local resistance data are important to be considered. On the other hand, non-antibiotic therapy has also become an option which should be discussed and offered to the patient. In patients suffering from recurrent uncomplicated cystitis, individual risk factors and possible behavioral changes should first be taken into account. Non-antimicrobial prophylactic strategies shown to be successful in well-designed clinical studies are the next options. Long term antibiotic prophylaxis, however, should only be considered as a last option. For some of those patients self-diagnosis and self-treatment may be suitable, e.g. by using a recognized questionnaire.

## 1 Epidemiology

Uncomplicated acute cystitis (uAC), also referred to as uncomplicated urinary tract infection (uUTI), is generally defined as an infection of the bladder in non-pregnant women with no known functional or anatomical abnormalities or co-morbidities [1]. These are distinguishable from acute pyelonephritis (an upper urinary tract infection) and complicated urinary tract infections (cUTI). The latter are a heterogeneous group of conditions and include those occurring in male and female patients with certain co-morbidities and abnormalities that impact urological function, and also include healthcare-associated and systemic infections [2].

It is well established that uUTIs are common in female patients of all ages, with an annual prevalence of ~11%, and are more common than cUTIs [2], [3]. Up to 80% of females will experience at least one uUTI in their lifetime, and as many as 45% will have recurrent uUTIs [4], [5], [6], [7]. Given their prevalence, uUTIs represents a substantial burden – without prompt and effective treatment, symptoms can be debilitating for several days and can impact work and daily routines [8], [9], [10].

The primary need of patients with uUTI is an accurate and early diagnosis followed by timely symptom relief. Current guidelines recommend empirical prescribing of selected antimicrobial agents [1], [11], [12], [13], [14], [15], which remains a largely effective approach for the acute episode. In young women experiencing a first episode of uUTI symptoms, urine culture is not recommended when a robust diagnosis can be reached by patient history-taking and other potential causes of symptoms can be excluded, which is important in order to minimize overdiagnosis and inappropriate treatment.

Indeed, uUTIs are one of the most common conditions associated with antimicrobial prescription [16], [17], and previous antibiotic exposure is associated with an increased risk of antimicrobial resistance (AMR), which may therefore present a public-health challenge [18], [19]. In particular, AMR of common uropathogens, e.g. *E. coli*, to therapies widely used for the management of uUTI, such as fluoroquinolones, is increasing in many regions [20]. Fluoroquinolones also transiently suppress commensal intestinal Enterobacteriaceae, associated with the development of AMR, and resistant strains can then spread to unexposed household contacts of patients treated with fluoroquinolones for urinary tract infection (UTI) [21]. Consequently, there is a need for novel oral therapies with activity against resistant strains of uropathogens, including extended-spectrum β-lactamase(-ESBL)-producing *E. coli* that are becoming more prevalent worldwide [22], [23], [24], [25], [26].

The safety of antimicrobial therapy is also a major concern. In recent years, the U.S. Food and Drug Administration (FDA) has published warnings regarding the use of fluoroquinolones for infections such as uAC [27], [28]. In particular, potential severe adverse effects on multiple organ systems, mentation and glucose control have resulted in recommendations that these drugs should not be prescribed for uAC unless there are no other alternatives [1], [11]. Therefore, alternative, non-antibiotic therapy for uAC should be considered and appropriate non-antibiotics or phytotherapeutics should be tested for equivalence with antibiotic therapy in well-designed clinical studies. Recurrent uUTIs are a major issue for many women and are associated with multiple visits to various healthcare professionals [29] and often repeated antibiotic prescriptions, with an increased risk of potential side effects [30], [31]. Some women may therefore prefer to avoid repeated courses of antibiotics and seek other treatment options [32].

An expert panel that included urologists, obstetricians/gynecologists, infectious diseases specialists, emergency medicine specialists, clinical microbiologists and primary care physicians representing a broad geographical spread (Europe, North America, Latin America and Asia) has summarized and discussed the different topics which still need to be investigated more carefully in order to help better the vast number of our patients suffering from acute episodes of uAC (Table 1 (Tab. 1)) [33].

## 2 Diagnosis of uncomplicated acute cystitis

Despite numerous publications, there is still no generally accepted strategy regarding the clinical diagnosis of uAC. The updated guidelines of the Infectious Diseases Society of America (IDSA) and the European Society for Microbiology and Infectious Diseases (ESCMID) mainly consist of recommendations about the treatment of uAC and not the diagnosis [11]. These guidelines were limited to the treatment of uAC and pyelonephritis in premenopausal, non-pregnant women with no known urological abnormalities or comorbidities. In addition, the authors noted that postmenopausal women or those who have well-controlled diabetes mellitus in the absence of urological sequelae may be considered as having uUTI by some experts, but a discussion of specific management of these groups was outside the scope of these guidelines.

In the last update of these guidelines of the European Association of Urology (EAU) from 2024, uAC is defined as acute, sporadic or recurrent cystitis limited to non-pregnant women with no known relevant anatomical and functional abnormalities within the urinary tract or comorbidities [1]. According to the EAU guidelines, the diagnosis of uAC can be made with a high probability based on a focused history of lower urinary tract symptoms (e.g. dysuria, frequency and urgency) and the absence of vaginal discharge or irritation.

The definition of UTIs in a broader sense is presented in the updated German National Clinical Practice S3 Guideline [34]: UTIs may be classified as uncomplicated in the absence of relevant functional or anatomical abnormalities in the urinary tract, with no relevant renal functional impairment and any relevant concomitant disease that could aggravate the UTIs or condition, which could increase the risk of development of serious complications. UAC in this regard may represent no additional health problem for the woman with stable diabetes mellitus, whereas any kind of pyelonephritis, whether earlier defined as uncomplicated or complicated, could interfere with her metabolic balance and could lead to severe complications. 

It becomes obvious today that a simple general classification of UTIs into uncomplicated and complicated UTIs is far too rough. Therefore, a more differentiated stratification of UTIs with deeper consideration of risk factors was proposed earlier [35].

The U.S. Food and Drug Administration (FDA) and European Medicines Agency (EMA) have proposed their guidelines for the clinical diagnosis of patients with uAC for clinical studies as follows:

- Adult and, if appropriate, adolescent females with evidence of pyuria (WBC≥10/μL) and at least two of the following signs or symptoms of dysuria, urinary frequency, urinary urgency, and suprapubic pain (FDA) [36]
- Female patients with documented pyuria (WBC≥10/μL) and a minimum number of symptoms such as frequency, urgency and dysuria (EMA) [37]

In both guidelines, FDA and EMA, for primary clinical outcome only patients with a so-called “significant” bacteriuria with ≥10^5^ colony forming units (CFU)/ml can be considered. However, significant bacteriuria of ≥10^5^ CFU/mL in adults was originally defined as significant only for the diagnosis of pyelonephritis [38]. In 1982, Stamm et al. [39] documented that the levels of ≥10^5^ CFU/mL of a pathogen in urine have a very high specificity (99%) but a very low sensitivity (51%) for the diagnosis of uAC. Bacteriuria of ≥10^2^ CFU/mL was suggested by the authors as the best diagnostic criterion (sensitivity, 95%; specificity, 85%). In 2013, Hooton et al. [40] confirmed that *E. coli* identified as low as 10^1^–10^2^ CFU/mL was sensitive and specific for the diagnosis of AC in symptomatic women. But still, about 20% of these symptomatic female patients were culture “negative” even when being tested for such low counts. Quantitative PCR (qPCR) for *E. coli* and *S. saprophyticus* finally demonstrated that almost all women with symptoms suggestive of UTIs and a “negative” culture still have an infection with *E. coli* [41].

We aimed to reassess first the diagnostic values of these proposed guidelines using the Acute Cystitis Symptom Score (ACSS), which was originally developed in Uzbek and Russian languages [42] and is now validated in several other languages as well (https://www.acss.world/). The ACSS diagnostic part A (Attachment 1) consists of 6 questions concerning typical symptoms, 5 questions concerning potential differential diagnosis, 3 questions concerning quality of life (QoL) and 5 additional questions about concomitant conditions such as female cycles and diabetes mellitus [43]. Each of the typical and differential symptoms and QoL questions can be answered according to 4 levels of severity (none, mild, moderate, severe) and the additional questions can only be answered with yes or no [43].

**Figure 1:**
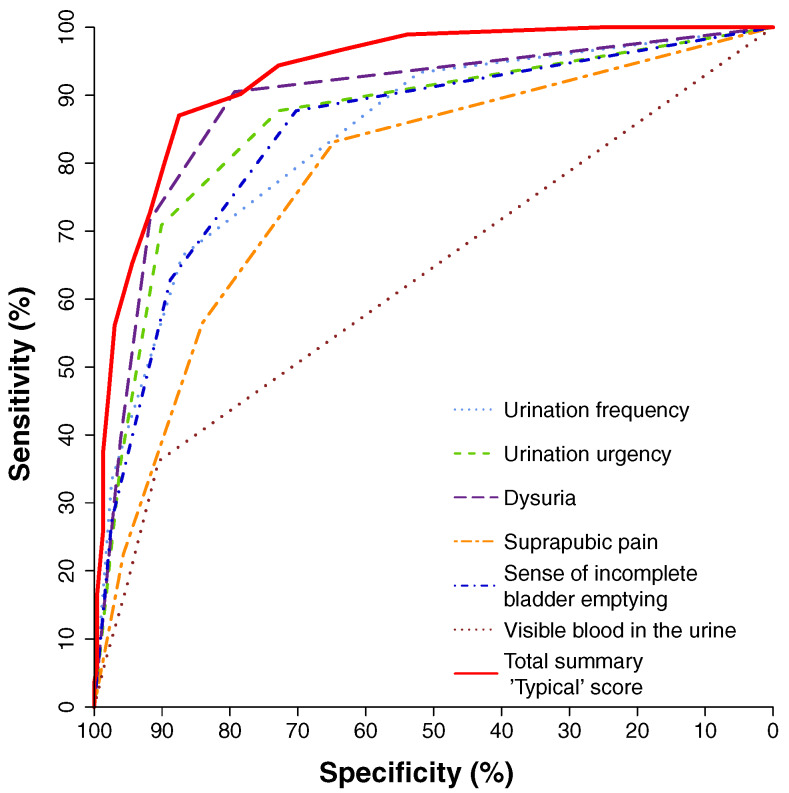
Receiver operating characteristic (ROC) curves for the six individual typical symptoms and the summary score of the six symptoms proposed by ACSS Source: Alidjanov et al. [44], licensed under CC BY 4.0 (https://creativecommons.org/licenses/by/4.0/)

In the analysis of the study, a total of 517 evaluable female respondents (285 patients with uAC with ages between 16 and 87 years and 232 controls without uAC with ages between 15 and 73 years) were included having used the ACSS in different languages: Uzbek (393), Tajik (65), German (43), Hungarian (16) [44]. The receiver operating characteristic (ROC) curves for the six individual typical symptoms and the summary score of the six symptoms proposed by ACSS showed that the best balance between sensitivity and specificity for the diagnosis of uAC can be reached if all 6 symptoms are considered together (Figure 1 (Fig. 1)). ROC curve analysis revealed the largest area under the curve (AUC) for the summary score of the “typical” domain of the ACSS (AUC [95% confidence interval (CI)]=0.93 [0.91; 0.95]), followed in descending order by dysuria (0.85 [0.82; 0.88]), urgency (0.85 [0.82; 0.88]), sense of incomplete bladder emptying (0.79 [0.75; 0.83]), suprapubic pain (0.74 [0.70; 0.78]), and visible blood in urine (0.63 [0.60; 0.67]).

**Figure 2:**
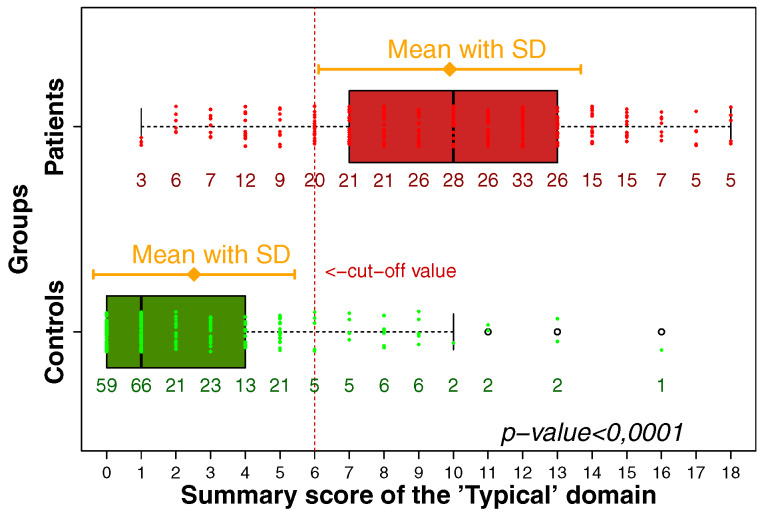
Boxplots (IQR, range, mean±SD) of the summary score of the six ACSS typical symptoms in patients with AC (n=285) and controls without AC (n=232) Source: Alidjanov et al. [44], licensed under CC BY 4.0 (https://creativecommons.org/licenses/by/4.0/)

Comparing only the 3 symptoms listed in the EMA guidelines with the 4 symptoms mentioned in the FDA guidelines and with the 6 symptoms mentioned in the ACSS, the sensitivity and specificity (average [95% CI]) for these different approaches of the diagnosis of uAC were (a) 0.84 [0.79; 0.88] and 0.83 [0.77; 0.87] for the EMA approach; (b) 0.83 [0.78; 0.87] and 0.88 [0.84; 0.92] for the FDA approach; and (c) 0.87 [0.83; 0.91] and 0.88 [0.83; 0.91] for the ACSS approach using a summary score of 6 and above as the cut-off, respectively (Figure 2 (Fig. 2)).

**Figure 3:**
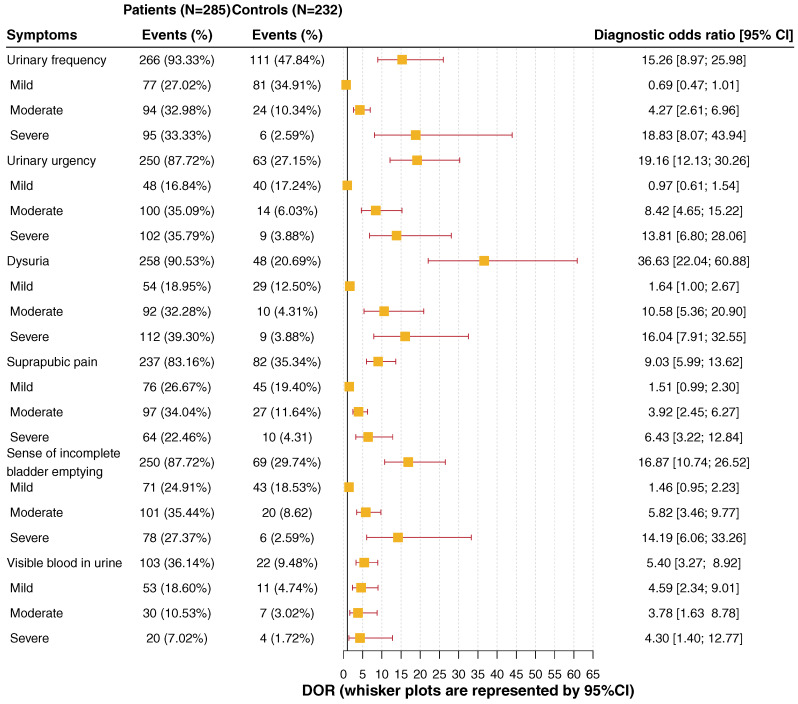
Prevalence and diagnostic odds ratio of the six ACSS typical symptoms Source: Alidjanov et al. [44], licensed under CC BY 4.0 (https://creativecommons.org/licenses/by/4.0/)

Therefore, it is also important to realize that not just the presence of a so-called typical symptom does differentiate significantly between patients with uAC and controls without uAC, but also the severity needs to be considered. Symptoms which are only considered mild do not differ significantly between the two patient groups (Figure 3 (Fig. 3)). 

### Bacterial isolates in female patients with uncomplicated acute cystitis

It is important to consider only those patients for analysis who also have the typical symptoms and the clinical diagnosis of uAC. Because of missing clinical data, laboratories often include all outpatients with UTI into the analysis who may suffer from different categories of UTI. It is also important to consider not only pathogens cultured in urine with CFU ≥10^5^/ml, but also bacteriuria with lower rates of CFU as mentioned earlier.

In a single-blind, randomized, multicentre study with 20 urologists in private practice, female patients aged 18 to 75 years with signs and symptoms of uAC were recruited to compare treatment of fosfomycin trometamol with ofloxacin and co-trimoxazole [45]. Midstream or catheter urine was collected for urine culture. The urine was also microscopically investigated for leucocytes and erythrocytes. At first visit of the 421 evaluable patients, 308 patients had a bacteriuria of ≥10^5^ CFU/ml, 79 had 10^2^–10^4^ CFU/ml, 21 had ≤10^1^ CFU/ml, and 13 had no culture performed. The distribution of bacterial isolates cultured in the 387 patients with ≥10^2^ CFU/ml are shown in Table 2 (Tab. 2), including 360 monoinfections and 27 mixed infections. *E. coli* was detected in 298 (77.0%) patients with monoinfections and 22 (5.7%) patients with mixed infections: a total of 320 (82.7%) patients (Table 2 (Tab. 2)).

This and many other studies have shown that *E. coli* is the most frequent and most important uropathogen for causing uAC. Therefore, according to the German National S3 Guideline, the detection of *E. coli* in symptomatic women is predictive for a bacterial UTI, irrespective of the number of pathogens. In contrast, the presence of *Enterococci* and group B *Streptococci* in urine is not predictive for UTIs and needs further investigations [34].

### Pathogenicity properties of uropathogen

Uropathogens such as *E. coli* must have specific pathogenic properties in order to trigger a clinically manifest infection. Bacterial attachment to the uroepithelium is the necessary initiating event permitting bacterial persistence, and also stimulates early activation of the innate immune system. 

Fimbriae/pili and nonfimbrial adhesins are responsible for adherence to the uroepithelium [46]. Type 1 fimbriated (fimH) *E. coli* strains are the predominant phenotypic variant isolated from patients with uUTI, and the presence of this adhesin is essential for establishing acute cystitis. Some of these adhesins (e.g. type 1 pili) also mediate internalization into the uroepithelium. Nests of intracellular persistent bacteria can be a source of recurrent infections [47].

Toxins such as alpha-hemolysin (α-Hly), cytotoxic necrotizing factor (CNF) or secreted autotransporter toxin (SAT) influence signal transduction mechanisms and modulate the host’s defense behavior. They also induce the death of uroepithelial cells (apoptosis, necrosis). Siderophores such as enterobactin, salmochelin, aerobactin and yersiniabactin bind with high affinity iron, which is an essential cofactor of many enzymes and therefore plays a role in the survival and growth of uropathogenic microorganisms [48].

The expression of these pathogenicity factors is strictly regulated and encoded by genetic determinants. This means that potential infectious agents can, on the one hand, adapt to host-specific defense functions and, on the other hand, trigger an infection if these are disrupted [48]. Local urinary cytokines regulate host defense against UTI. Activation of Toll-like receptors on uroepithelial cells promotes release of cytokines which, in turn, recruit and activate granulocytes, macrophages, monocytes and other immune regulatory cells [49].

## 3 Antibiotic treatment of uncomplicated acute cystitis

In most guidelines so far antibiotic therapy of the acute episode of uAC is mentioned in first place [1], [11], [12], [13], [15], [34], [50], [51], [52]. Therefore, at best the local antibiotic resistance especially of *E. coli*, the most frequent and important uropathogen causing uAC, should be known, which may differ between countries and areas.

Real-world data were collected in five different areas of Germany by physicians from three medical specialities (urology, general medicine/internal medicine/primary care, and obstetrics/gynecology) to examine antimicrobial resistance (AMR) prevalence, treatment patterns, and clinical outcomes among female patients with uUTI [53]. Data were collected from a retrospective physician-based chart review completed by physicians treating patients with uUTI. Non-pregnant women aged ≥12 years, with a uUTI diagnosis, an *E. coli*-positive urine culture between January 2017 and December 2019, and susceptibility test results according to EUCAST breakpoints for at least ≥4 drug classes were eligible, while “susceptible at higher dose/susceptible + intermediate” was used for analysis.

Patients were stratified into three cohorts by *E. coli* susceptibility to six drug classes (fosfomycin; nitrofurantoin; mecillinam; fluoroquinolones including ciprofloxacin, levofloxacin, and ofloxacin; cefpodoxime; folate metabolism inhibitors (FMIs) including trimethoprim and trimethoprim-sulfamethoxazole): susceptible to all (SUS), resistant to one or two drug classes (DR1/2), and resistant to ≥3 (MDR) drug classes tested. Among 386 eligible patients [SUS (67.1%); DR1/2 (29.0%); MDR (3.9%)], AMR prevalence was FMIs (18.3%) and lowest for fluoroquinolones (5.2%). The drugs prescribed most often were fosfomycin in SUS (44.0%), DR1/2 (41.4%), and fluoroquinolones in MDR (40.0%) (Table 3 (Tab. 3)). Between the 5 regions in Germany, one could observe some differences in the rate of resistance of more than 10% for mecillinam and the fluoroquinolones. The resistance rates for trimethoprim or cotrimoxazole were in the range around 20% (17.5%–26.1%) except in the southwest with only 12.7%, whereas in the northeast and in the southeast the resistance rates were >20% (Table 4 (Tab. 4)).

Treatment for uUTI failed for 8.8% of patients; failure was more likely in MDR versus SUS [adjusted odds ratio [95% CI]=4.21 [1.14–1.50]; P=0.031); incidence of recurrent infection in the 6-months post-index period was higher in DR1/2 versus SUS. These findings may have implications for empiric prescribing, suggesting an unmet need for new treatments [53].

In the updated EAU guidelines 2024, the first line oral antibiotics recommended for treatment of uAC in women are fosfomycin trometamol, nitrofurantoin, pivmecillinam, alternatively oral cephalosporins, e.g. during pregnancy. Trimethoprim or cotrimoxazole are only recommended if the local resistance pattern for *E. coli* is <20%, which e.g. in Germany is not anymore the case everywhere [1]. The German guidelines [54] also recommend in addition nitroxoline as a first line oral antibiotic for treatment of uAC (Table 5 (Tab. 5)), which is available in 9 European countries (Bulgaria, Croatia, Germany, Georgia, Lithuania, Poland, Romania, Russia and Serbia). In a phase 3 study, the efficacy of nitroxoline was non-inferior with cotrimoxazole and ofloxacin (95% confidence interval <10%) and a recent microbiological study in Germany showed that *E. coli* from uAC were 100% susceptible to nitroxoline [55], [56].

## 4 Alternative management, non-antibiotic therapy of uncomplicated acute cystitis in women

Already in 2012, the American infectiologist T.M. Hooton rightly assessed antibiotic prescriptions for uUTI very critically [57]. Around 25% of all antibiotic prescriptions are for uUTI, although many such conditions are self-limiting or could be treated non-antibiotically and therefore antibiotics are not always necessary. This approach limits the development of resistance, reduces drug costs, influences the risk of collateral damage and improves the compliance of affected individuals.

A symptom-oriented treatment with non-steroidal analgesics/anti-inflammatory drugs has been investigated in several studies. This showed a significant reduction in the use of antibiotics [58], [59], [60]. However, with this strategy the symptoms lasted slightly longer and slightly more cases of pyelonephritis were observed, although the recurrence rate was not increased after symptomatic therapy as compared to antibiotic therapy [61], [62], [63].

The treatment of uAC with herbal preparations, for example with BNO 1045 (Canephron: lovage root, rosemary leaves, centaury herb), showed non-inferiority in terms of symptom duration compared to antibiotic therapy with fosfomycin [64]. This study also showed that the local immune reaction due to the infection could be normalized through therapy with this phytotherapeutic agent [65]. Therefore, non-antibiotic therapeutic measures should be considered as a treatment option for uUTIs which should be discussed with the patient [54].

### Patient-reported outcome

In clinical studies with non-antibiotic therapy, but also in so called non-interventional studies (NIS), in which usually no urine cultures are performed, the elimination of bacteriuria cannot be the therapeutic aim. Therefore, the clinical outcome is much more important, for which we need standardized measures. The 2^nd^ part of the ACSS is suitable as a patient-reported outcome measure (PROM), which was developed in this international study mentioned earlier as part 2 [44], [66]. 134 patients of among 517 previously selected female respondents [44] were included into the patient-reported outcome (PRO) analysis. The age of the selected patients ranged from 17 to 82 years, with a median (IQR) of 31 (24.00–44.25) and mean (SD) of 36.28 (16.03) years. Of these, 109 filled out at least 1 copy of the “follow-up Part B” form of the ACSS (Attachment 1) (one “follow-up” visit) after the initial “diagnostic” visit and 25 patients filled out multiple copies at different “follow-up” visits. Altogether, they have formed 236 cases.

**Figure 4:**
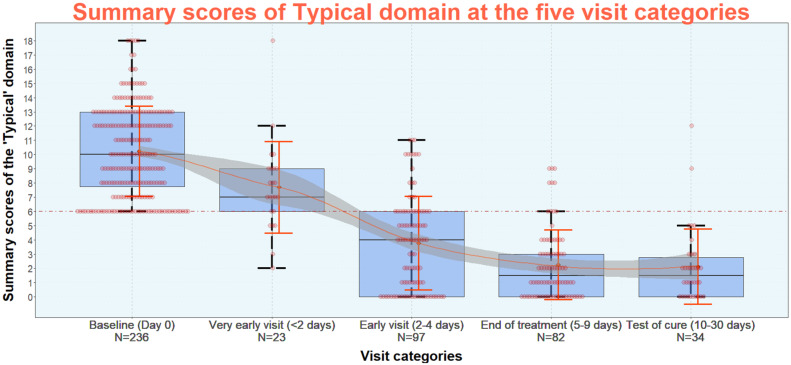
Summary scores of the six typical symptoms (“Typical” domain) of the ACSS at diagnostics of acute uncomplicated cystitis (AC) in women (baseline) and at the four different follow-up visit categories Source: Alidjanov et al. [66], licensed under CC BY 4.0 (https://creativecommons.org/licenses/by/4.0/)

Figure 4 (Fig. 4) shows the reduction of the summary scores of the six typical symptoms (“Typical” domain) of the ACSS at diagnostics of uAC in women (baseline) and at the four different follow-up visit categories. At the visits “end of treatment” and “test of cure”, most of the women had a summary score of the typical symptoms <6. From further analysis a summary score of the “Typical” domain up to a maximum of 5 with no symptom >1 (mild) AND no visible blood in the urine was recommended as threshold for successful clinical outcome for further studies. Using this threshold the treatment was clinically successful at end of treatment in 80.5% and at test of cure in 80.4%. As secondary outcome a summary score of the three Quality of Life (QoL) categories up to 3 with no item >1 and a score of no more than 1 for the overall outcome (Dynamics) listed on the head of the ACSS part B (Attachment 1) should be considered [66].

Although reports of patients concerning symptoms can only be subjective by definition, by answering the same, in the meantime familiar questionnaire at any follow-up visit, one can at least expect that by scoring the symptoms not only the presence or absence, but also the increasing or decreasing severity of each symptom reported by the patient can be considered as a quasi-objective measure.

Since non-antibiotic therapy has become an alternative approach to treat uAC in women, suitable PROMs are urgently needed. Although typical symptoms are mainly used for clinical diagnosis and outcome, these symptoms are not exclusively found in uAC. Therefore, severity scoring of the symptoms is needed not only for diagnostics, but also for PROM to define “clinical cure” of any intervention. The presented data analysis demonstrated that the ACSS has the potential to be used as a suitable instrument for PROM in well-designed prospective clinical studies [66].

A systematic literature review revealed 23 studies reporting on six different PROMs for uUTI in women. From those, the Acute Cystitis Symptom Score (ACSS) and the Urinary Tract Infection-Symptom and Impairment Questionnaire (UTI-SIQ-8) were recommended for further use. Both instruments showed sufficient content validity [67].

## 5 Recurrent uncomplicated cystitis in women

The current European Association of Urology (EAU) guidelines define recurrent urinary tract infections (UTIs) as recurrences with a frequency of at least three UTIs in the past year, or two UTIs in the last 6 months [1]. Risk factors for recurrent UTI are discussed in depth by Cai [68]. The principal risk factor in sexually active pre-menopausal women is frequency of sex [68], [69]. Other behaviors including use of spermicide, having a new sexual partner within the past year, pre-/post-coital voiding habits, delayed voiding habits/periodicity of urination and vaginal douching also affect risk of recurrence [68], [69]. In addition, early onset (<15 years old), family history, body-mass index and urine voiding disorders all increase risk in younger women [68], [69]. Major risk factors in older women appear to be substantially related to the effects of reduced estrogen levels and include atrophic vaginitis, cystocele, increased post-void urine volume and functional status deterioration [70], [71]. Cai et al. have analysed the risk of UTI recurrence for various factors and created a nomogram for the calculation of the overall risk for UTI recurrence which has substantial clinical utility (Table 6 (Tab. 6)) [72].

The relationship between acute symptomatic UTI and reduced quality of life (QoL) has been well established for some years [72], [73]. Naber et al. [74] performed a PubMed/MEDLINE search on literature from 2000 to 2020 using the terms (“recurrent UTI” OR “recurrent urinary tract infection”) AND (“anxiety” OR “depression” OR “quality of life”) in order to identify any recent high-quality meta-analyses or systematic reviews as well as other relevant publications and found ten studies appropriate for their review.

In one such study using data from a large UK Internet self-help forum hosted by a charity supporting people with bladder problems (N=5,994), the authors highlighted patient descriptions of reduced quality of both intimate and social relationships, self-esteem, and capacity for work due to recurrent UTI [75]. Women also frequently described broader systemic disabling symptoms than those typically ascribed to UTI, including flu-like symptoms, spasms, and both back and leg pain [75]. In addition, seemingly mild symptoms such as increased frequency and urgency of urination were discussed in terms of their anxiety-inducing effects and disruption to sleep patterns with the potential to cause persistent fatigue [75]. One contributor stated: “I find that this affects every aspect of my life”.

The European GESPRIT (GErmany, Switzerland, Poland, Russia and ITaly) study used a self-administered online survey which assessed course of disease; social and economic burden; disease management and QoL effects (SF-12v2 questionnaire) related to recurrent UTI [29]. The study included adult women who had suffered from recurrent UTI and who were currently affected by an acute UTI or had experienced an episode within the 4 weeks prior to entering the study. Approximately three days of sick leave were taken per year across the full study population (2.3 days in Switzerland to 3.9 days in Germany). Limitations to daily activity occurred on approximately 3.5 days per year in the full population (2.6 in Poland to 4.0 days in Russia) [30]. The mean number of doctor visits per year was 2.8, ranging from 1.7 visits in Russia to 3.7 visits in Germany (P<0.0001).

For the mental components of SF-12v2, the most significant reduction overall was in mental role functioning, followed by the previously mentioned social functioning and mental health, which both reduced by a similar extent [29]. With the exception of vitality, all mental-health components were reduced by a similar or greater extent than physical components, and the overall mental score was substantially lower. Of the 90% of women who answered questions related to sexual function (n=1,745), 34% suffered from UTI very often or often after sexual intercourse, with a substantially higher proportion of patients (57%) stating that sexual relations were negatively influenced by UTI [29].

### Specific aspects of microbiology in recurrent uncomplicated cystitis

In female patients with recurrent uAC, the distribution of uropathogens is about the same as for patients with sporadic cystitis during the acute episode (Table 2 (Tab. 2)) [45]. Asymptomatic bacteriuria (ABU) between the acute episodes, however, has shown not to be an additional risk factor for recurrent UTI. In contrast, ABU may even reduce the number of recurrences as has been shown [76]. Therefore, ABU should also not be treated in patients with recurrent UTI. 

In another study, 20 patients were randomized to blinded inoculations with *E. coli* 83972 or saline into the bladder [77]. In phase 1, the time to the first UTI was longer with than without *E. coli* 83972 bacteriuria (median 11.3 vs 5.7 months, sign test p=0.0129). Phase 2 was analyzed after patients had spent a total of 202 months with and 168 months without *E. coli* 83972 bacteriuria. There were fewer reported UTI episodes with vs without *E. coli* 83972 bacteriuria (13 vs 35 episodes, paired t test p=0.009, CI 0.31–1.89). There was no febrile UTI episode in either of the study arms and no significant side effects of intravesical bacterial inoculation were reported [77].

On the other hand, the uropathogens causing recurrent episodes of UTI may also stay in the bladder environment between the episodes. Naboka et al. [78], [79] investigated midstream urine samples taken from 169 women between episodes of recurrent lower UTI (LUTI) and analyzed the strains for virulence factor genes (VFGs) often grouped into clusters called “pathogenicity islands” because the pathogenic potential of microorganisms depends on the presence and/or appearance of their specific properties to interact with the host. Sixty-two strains of Enterobacteriaceae at concentrations 10^2^–10^8^ CFU/ml were analyzed for the presence of the following VFGs:

**Adhesive structures coding:**

P-pili group:

- papA is the structural subunit of P pili (colonization factor in extraintestinal infections)
- papE/F – apical adhesion of P pili
- papGII – apical adhesion of P pili (II allele)
- afa – afimbrial adhesin (adhesin binds to the DAF receptor on the epithelium of the cell surface, also provides the ability to hemagglutination)
- bmaE – M pili

**Coding of iron absorption systems:**

- fyuA, iutA, feoB – synthesis of siderophores

**General pathogenicity:**

- kpsMII – capsule synthesis
- usp – uropathogenic specific protein

In all strains VFGs were found with numbers from 1 to 10. Four VFGs were found at all levels of bacteriuria (from 10^2^ to 10^8^) in most strains (>50%): papGII, feoB, fyuA and usp. Each of the genes papA, papE/F and usp was found more often in uropathogens from patients with a higher level of leukocyturia. The authors concluded that the inter-episode period in recurrent LUTI is associated with varying levels of bacteriuria of enterobacteria. Since enterobacteria virulent potential could be determined at all levels of bacteriuria, there is a potential risk for recurrence of LUTI at all levels of bacteriuria.

### Prophylaxis of recurrent uncomplicated cystitis in women

According to the German guidelines, antibiotics should not be used primarily for the prophylaxis of recurrent uUTIs [54]. For prophylaxis, sufficient drinking quantities are sometimes sufficient, which should be at least 1.5 liters per day [80]. Regarding the value of cranberries in preventing recurrent UTIs, Jepson et al. found in their Cochrane review 2012 that cranberry products did not significantly reduce the incidence of symptomatic UTI overall or in women with recurrent UTI compared to placebo, water or no treatment [81]. However, recent systematic reviews including meta-analyses were able to prove that consumption of mono- or combination products containing cranberries had indeed a positive preventive effect on the rate of UTI recurrences in otherwise healthy women [82], [83], [84], [85], [86], [87]. This result was also confirmed in the most recent Cochrane review 2023 [88].

The different study results may be due to the clinical and methodological heterogeneity of the included studies. One explanation for this would be, for example, the different content of proanthocyanidin (PAC) in the different products. Cranberry has been shown to inhibit the adhesion of uropathogenic *E. coli* to uroepithelial cells, which is caused by PAC in vitro in a dose-dependent relationship [89], [90], [91]. Since the data currently available for comparison with long-term antibiotic prevention are contradictory, according to the EAU guidelines there is only a weak recommendation to use cranberry to prevent recurrent UTIs [1]. Other phytotherapeutics should also be discussed, e.g. mustard oils from watercress and horseradish [92].

The use of D-mannose to treat uUTIs is based on insights into the process of bacterial cytoadherence. D-mannose has structural similarities to those glycoproteins. Uroepithelium (uroplakin) binds to the *E. coli* via their type 1 fimbriae. This means that D-mannose can competitively inhibit the adhesion of infectious pathogens in the urinary tract [93]. A prospective, randomized, clinical study has shown that this strategy may be an alternative to the use of antibiotics in women with uncomplicated recurrent UTI [94].

Two systematic reviews including a meta-analysis examined the effect of D-mannose on the rate of recurrent UTI. Lenger et al. analyzed data from 390 patients and came to the conclusion that D-mannose is effective in preventing recurrent urinary tract infections compared to placebo and has comparable effectiveness to antibiotic prophylaxis [95]. D-mannose was well tolerated, with only 8/103 (7.8%) patients complaining of diarrhea. In another systematic review with 695 patients, D-mannose was shown to improve quality of life, significantly reduce recurrent urinary tract infections in both catheter users and non-catheterized patients, reduce the incidence of rUTIs and prolong the recurrence-free time [96]. However, a current systematic Cochrane review, in which a total of 719 patient data were analyzed, was unable to determine whether D-mannose significantly reduces the number of recurrent urinary tract infections compared to no treatment, other dietary supplements or antibiotics [97].

Immunomodulation is another option for prevention of recurrent UTI. The goal is to activate specific immunity to improve humoral and cell-mediated innate defense mechanisms in the host organism. The first available agent, OM-89, is administered orally for 3 months and boostered in the 7^th^ to 9^th^ month for 10 days each month. It contains lyophilized bacterial lysates from 18 uropathogenic *E. coli* strains. In vitro it was effective in stimulating the metabolism of murine spleen cells within a concentration range of 0.625–2.5 mg/ml. The activation of murine bone marrow-derived macrophages by OM-89 was shown by the induction of NO production. In the human system, the effect of OM-89 was tested in vitro: metabolic activity of peripheral blood lymphocytes (PBL) was stimulated starting at concentrations of approx. 250 microg/ml, and the spontaneous apoptosis of polymorphonuclear neutrophils (PMN) was reduced starting at OM-89 concentrations of approx. 100 microg/ml [98]. In three meta-analyses of the five prospective, randomized, placebo-controlled clinical studies between 1990 and 2005 including about 1,000 adult patients with an observation period of 6 (four studies) to 12 (one study) months, the number of UTIs was significantly lower in OM-89-treated patients (mean 39%), as was the use of antibacterials [82], [83], [99], [100].

Urovac is another such possibility which is available as vaginal suppository. It consists of 10 strains of heat-killed uropathogens: six from *E. coli *of different serotypes and one each from* Klebsiella pneumoniae*, *Proteus vulgaris*, *Morganella morganii* and *Enterococcus faecalis*. In the four meta-analyses mentioned earlier [82], [83], [99], [100] three randomized clinical trials (RCT) could be analyzed. Primary immunization consisted of 3 vaginal vaccine suppositories at weekly intervals (with 1 [low dose] or 2 [high dose] ampules of 2x10^9^ heat-killed organisms per suppository). In 2 of the 3 studies the addition of booster vaccination was evaluated. Booster immunization consisted of 3 additional vaccine suppositories (with 1x10^9^ killed organisms or 2x10^9^ killed organisms) at monthly intervals. The time until first reinfection, the proportion of women experiencing UTI and the mean number of UTIs during follow-up were all in favor of the booster immunization group compared to those receiving placebo or primary immunization only. The comparison of all the studies above was limited by the nature of definition of UTIs, and the duration over which the interventions were assessed against placebo.

A further option could be the intramuscular StroVac consisting also of 10 strains of heat-killed uropathogens: six from *E. coli* of different serotypes and one each from *Klebsiella pneumoniae*, *Proteus mirabilis*, *Morganella morganii* and *Enterococcus faecalis*. It should be injected three times at weekly intervals and boostered with one injection 12 months later. Its efficacy of prophylaxis for recurrent UTI was determined in a two-year follow-up study in women with recurrent UTI with StroVac (n=124) compared to Nitrofurantoin (n=49) 100 mg once daily over three months. In the first 12 months, 86.8% of patients in the StroVac group and 91.8% in Nitrofurantoin group were successful (p=0.22). Side effects were noted in 2.3% in the StroVac group causing discontinuation of therapy, whereas in the Nitrofurantoin group 18.4% stopped medication premature, mostly due to mild diarrhoea. In the second year, 79.3% of patients in the StroVac group were still successful, most of them had undergone booster injection. In contrast, in the Nitrofurantoin group only 59.2% of patients were still successful (p=0.03). Successful vaccination was defined as one or fewer UTIs in 12 months following vaccination and booster injection, respectively [101]. 

In a recent placebo controlled randomized study, however, 376 patients were randomized. In the StroVac group (n=188), the number of UTIs was reduced from 5.5 to 1.2, in the placebo group (n=188) from 5.4 to 1.3 (p=0.63). StroVac reduced the number of clinically relevant UTIs similar as placebo and did not show statistically significant better results than the placebo [102].

MV140, the most recent option administered as a sublingual spray, is a glycerinated suspension of whole-cell, heat-inactivated bacteria, including equal amounts of four common UTI-causing pathogens: *Escherichia coli*; *Klebsiella pneumoniae*; *Proteus vulgaris*; and *Enterococcus faecalis* (MV140 formulation) [103]. Since MV140 has shown excellent long-term effectiveness in previous observational studies after 3-month daily administration [103], in a multicenter, randomized, double-blind, placebo-controlled, parallel group 1-year trial, women with recurrent UTI were allocated to receive MV140 for 3 or 6 months or placebo for 6 months in a 1:1:1 ratio. The median (interquartile range) of UTI episodes was 3.0 (0.5 to 6.0) for placebo compared with 0.0 (0.0 to 1.0) in both groups receiving MV140 (P<0.001). Among women treated with placebo, 25% (95% confidence interval [CI], 15% to 35%) were free of UTIs compared with 56% (95% CI, 44% to 67%) and 58% (95% CI, 44% to 67%) of women who received 3 and 6 months of MV140 treatment, respectively [104].

For postmenopausal women with recurrent UTI, topically applied intravaginal estriol cream has shown good results in a randomized, double-blind, placebo-controlled trial [71]. The incidence of UTI in the group given estriol was significantly reduced and more of the women in the estriol group than in the placebo group remained free of UTI during the observational period of 8 months. Lactobacilli were absent in all vaginal cultures before treatment and reappeared after one month in 61% estriol-treated women but in none of the placebo recipients (P<0.001). With estriol the mean vaginal pH declined from 5.5 to 3.8 (P<0.001), whereas there was no significant change with placebo. The authors suggested that the intravaginal administration of estriol prevents recurrent UTI in postmenopausal women, probably by modifying the vaginal flora [71]. Although vaginal estrogens reduced the number of UTIs, oral estrogens did not. In addition, oral estrogens are associated with coronary heart disease, venous thromboembolism, stroke, and breast cancer. Therefore, oral estrogens are not recommended in postmenopausal women to prevent recurrent UTIs [82], [83].

Another option is the use of lactobacilli preparations because specific lactobacilli strains seem to have the ability to interfere with the adherence, growth, and colonization of uropathogenic bacteria [105]. In a double-blind placebo-controlled trial of a *Lactobacillus crispatus* intravaginal suppository probiotic (Lactin-V; Osel) for prevention of recurrent UTI in premenopausal women high-level vaginal colonization with *L. crispatus* (≥10^6^ 16S RNA gene copies per swab) throughout follow-up was associated with a significant reduction in recurrent UTI only for Lactin-V receiving women (P<0.01) [106]. In other studies, different lactobacilli and different preparations were also used with ambiguous success, e.g. vaginal suppositories containing *L. casei v rhamnosus* [107] or drinks containing *Lactobacillus GG* [108] or capsules containing *L. rhamnosus* GR-1 and *L. reuteri* RC-14 [109].

L-methionine is a sulfur donor necessary for the biosynthesis of cysteine, a sulfide amino acid. The action of L-methionine achieves a physiological pH value that creates unfavorable conditions for bacterial colonization, since a large number of Gram-negative bacteria are able to alkalize the urine through enzymatic breakdown of urea [110]. Cai et al. evaluated the efficacy of a phytotherapeutic combination of L-Methionine associated with Hibiscus sabdariffa and Boswellia serrata for treatment of acute episodes of uncomplicated UTI in women affected by recurrent UTIs in comparison to antibiotic treatment and demonstrated that this phytotherapeutic combination was able, in comparison to antibiotic treatment, to improve patients’ quality of life, reducing symptoms in acute setting and preventing the recurrences. A significantly higher proportion of patients in the phytotherapy group had asymptomatic bacteriuria (ASB) after three months [110]. This may be of interest because this group also showed that ASB should not be treated in young women affected by UTI because it may play a protective role in preventing symptomatic recurrence [76]. These data have been confirmed by a systematic review and meta-analysis, published in 2021, demonstrating that medical device containing xyloglucan, hibiscus and propolis is superior to comparator regimens in terms of clinical effectiveness in adult women with microbiologically confirmed or clinical suspicion of uncomplicated cystitis and is associated with a high patient compliance [111].

Pentosan polysulfate sodium (PPS) is an oral medication approved by the U.S. Food and Drug Administration for the treatment of painful bladder syndrome/interstitial cystitis to manage pain, urgency, and frequency of urination [112]. Similar to heparin, PPS is a semi-synthetic polysaccharide which has anticoagulant effects. After being excreted to urine, PPS provides a protective coating to the damaged urothelium, restoring the integrity of GAG layer, and decreases the permeability of the urothelium [113]. Tseng et al. [114] conducted a prospective multicenter randomized open-label trial to investigate the efficacy and safety of PPS for prevention of recurrent UTI in women and could demonstrate that a 16-week PPS monotherapy significantly reduced UTI recurrence when compared with an observational group as control.

In a recent multicentre, open label, randomised, non-inferiority trial [115] for prophylaxis of recurrent UTI, methamine hippurate was compared with antibiotic prophylaxis. All participants were observed for at least six months. The modified intention-to-treat analysis comprised 205 (85%) participants (antibiotics, n=102 (85%); methenamine hippurate, n=103 (86%)). Incidence of antibiotic-treated urinary tract infections during the 12 month treatment period was 0.89 episodes per person year (95% confidence interval 0.65 to 1.12) in the antibiotics group and 1.38 (1.05 to 1.72) in the methenamine hippurate group, with an absolute difference of 0.49 (90% confidence interval 0.15 to 0.84) confirming non-inferiority. Adverse reactions were reported by 34/142 (24%) in the antibiotic group and 35/127 (28%) in the methenamine group and most reactions were mild. Therefore, the recent EAU guidelines [1] made a strong recommendation for methemanine hippurate.

According to the EAU guidelines [1], use of endovesical instillations of hyaluronic acid or combinations of hyaluronic acid and chondroitin sulphate to prevent recurrent UTI has only a weak recommendation. Patients should be informed that further studies are needed to confirm the results of initial studies [116], [117], [118].

## 6 Conclusions

Since uAC is a very frequent infection in women involving several medical specialities, like urology, gynecology, and general medicine, it is important to develop common therapeutic strategies feasible and acceptable not only for daily practice, but also for clinical studies. Although antibiotic therapy of the acute episode and prophylaxis of recurrent UTI may still be an important pillar to be considered as a last resort, non-antibiotic therapy and prophylaxis should be propagated and investigated much further to lower antibiotic usage in general to reduce not only possible adverse events, but in particular selection of antibiotic resistant uropathogens which may be able to cause severe pyelonephritis or even urosepsis in some cases, for which effective antibiotic therapy is absolutely necessary.

## Note

This article is also to be published as a chapter of the Living Handbook “Urogenital Infections and Inflammations” [119].

## Dedication

This publication is dedicated to Professor Karl-Horst Bichler for his 90^th^ birthday.

## Copyright of the ACSS

The ACSS is copyrighted by the Certificate of Deposit of Intellectual Property in Fundamental Library of Academy of Sciences of the Republic of Uzbekistan, Tashkent (Registration number 2463; 26 August 2015) and the Certificate of the International Online Copyright Office, European Depository, Berlin, Germany (Nr. EU-01-000764; 21 October 2015). The rightsholders are Jakhongir Fatikhovich Alidjanov (Uzbekistan), Ozoda Takhirovna Alidjanova (Uzbekistan), Adrian Martin Erich Pilatz (Germany), Kurt Guenther Naber (Germany), and Florian Martin Erich Wagenlehner (Germany). The e-USQOLAT is copyrighted by the Authorship Certificate of the International Online Copyright Office, European Depository, Berlin, Germany (Nr. EC-01-001179; 18 May 2017). Translations of the ACSS in other languages are available on the website https://www.acss.world/downloads.html.

## Competing interests

KGN, JA, AP, and FMW are authors and copyright holders of the ACSS questionnaire. KGN is a consultant of Adamed Pharma, Bionorica, BioMerieux, GlaxoSmithKline, Immunotek, Ingenion Medical, Janssen Pharmaceutica, OM Pharma, and MIP Pharma. JA is an employee of Bionorica SE. WLS is a consultant of Bionorica, Desitin, MIP Pharma. JK is a consultant of Bionorica and GSK. FMW is a consultant of Achaogen, Astellas, AstraZeneca, Bionorica, MSD, Eumedica, GSK, Janssen, Klosterfrau, MIP Pharma, Pfizer, OM Pharma, Qiagen, VenatoRx. RF declares no conflict of interest.

## Supplementary Material

American English Acute Cystitis Symptom Score (ACSS)

## Figures and Tables

**Table 1:**
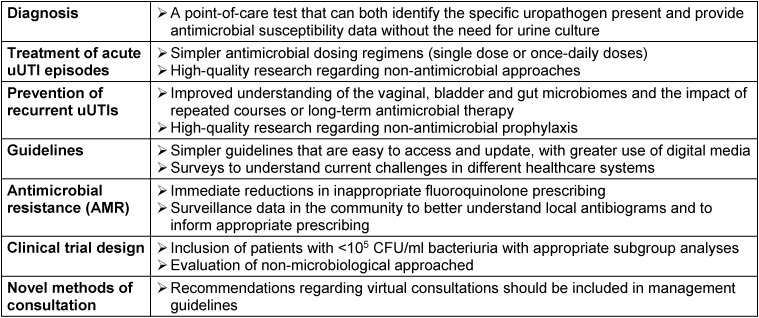
Summary of unmet needs in the diagnosis and management of uncomplicated urinary tract infections (uUTIs) Table by Wagenlehner et al. [33], licensed under CC BY 4.0 (https://creativecommons.org/licenses/by/4.0/)

**Table 2:**
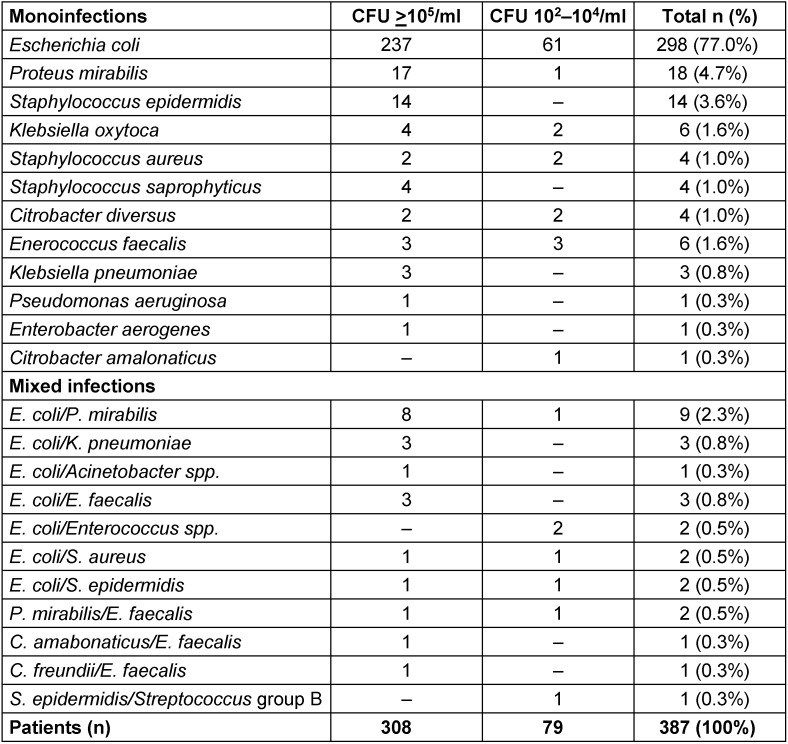
Urinary bacterial isolates at first visit in 387 female patients with uAC Table adapted from Naber et al. [45]

**Table 3:**
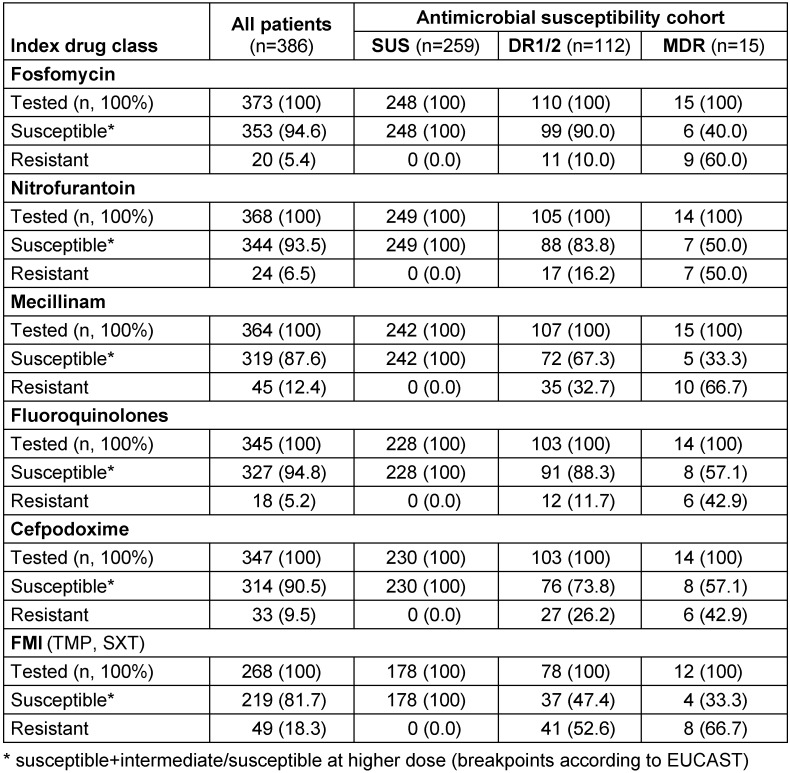
Antimicrobial resistance among non-pregnant female patients with uncomplicated UTI who had bacterial susceptibility data for ≥4 antibiotic drug classes: fosfomycin; nitrofurantoin; mecillinam; fluoroquinolones including ciprofoxacin, levofoxacin, and ofloxacin; and folate metabolism inhibitor (FMI) including trimethoprim (TMP) and trimethoprim-sulfamethoxazole (SXT). SUS – susceptible to all drug classes, DR1/2 – resistant to one or two drug classes, MDR – resistant to three or more drug classes tested Table adapted from Naber et al. [53], licensed under CC BY 4.0 (https://creativecommons.org/licenses/by/4.0/)

**Table 4:**
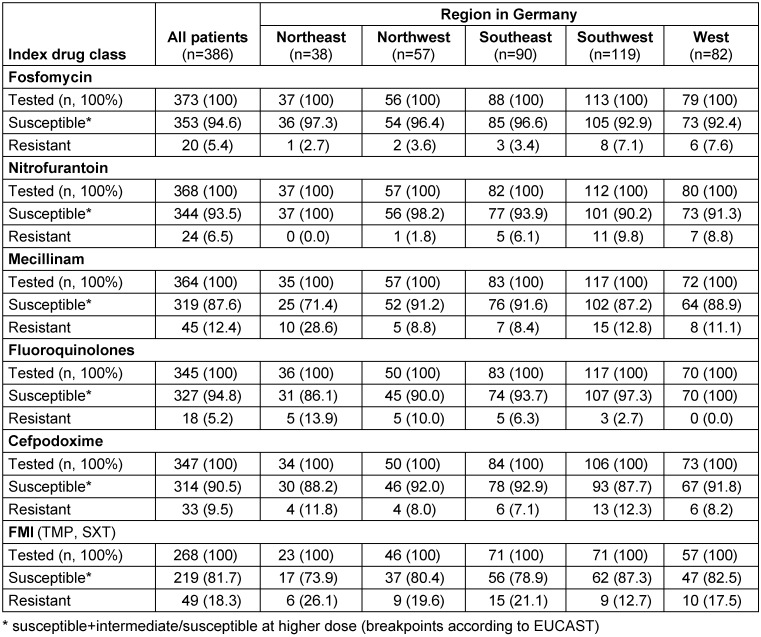
Antimicrobial resistance among non-pregnant female patients with uncomplicated UTI who had bacterial susceptibility data for ≥4 antibiotic drug classes (s. Table 3), stratified by regions in Germany Table adapted from Naber et al. [53], licensed under CC BY 4.0 (https://creativecommons.org/licenses/by/4.0/)

**Table 5:**
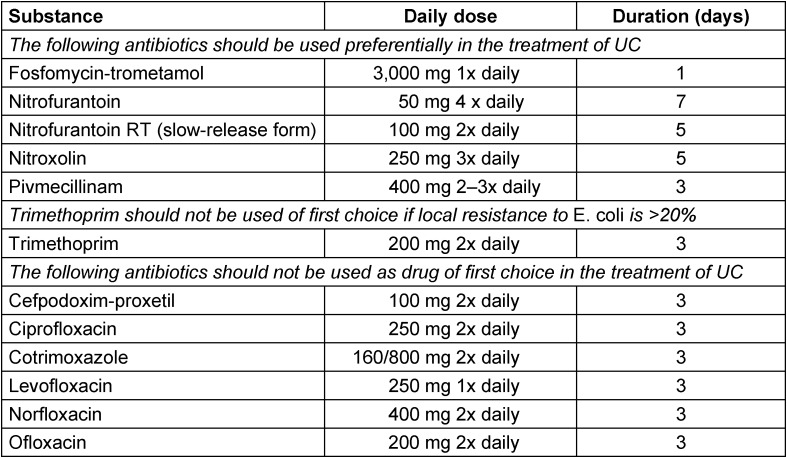
Recommended empirical short-term antibiotic treatment of uncomplicated cystitis (UC) in women in the premenopause (standard group; listing in alphabetical order) Table adapted from Kranz et al. [54]

**Table 6:**
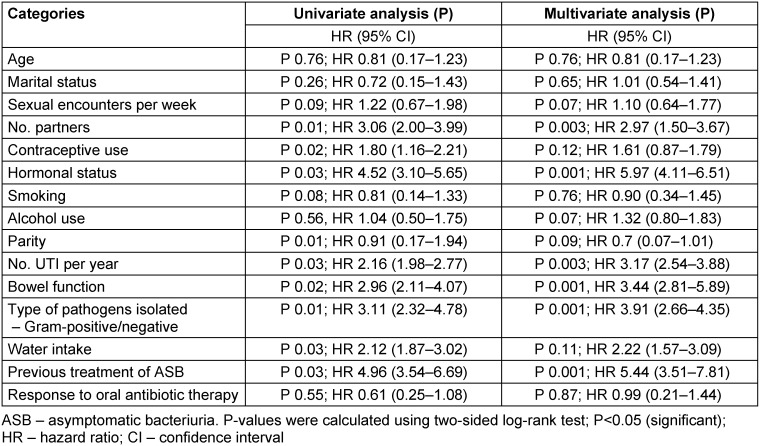
Univariate and multivariate analysis results of factors affecting recurrence-free in 768 patients enrolled in the training set according to Cai et al. [72] Table adapted from Cai et al. [72]
