# Supplementary material for: Therapeutic strategies for uncomplicated cystitis in women
Source: GMS Infect Dis. 2024 Apr 29;12:Doc01. doi: 10.3205/id000086 (PMC11099625; doi:10.3205/id000086)
Supplement: American English Acute Cystitis Symptom Score (ACSS) [file ID-12-01-s-001.pdf]

Attachment 1: American English Acute Cystitis Symptom Score (ACSS)

Adapted from: Alidjanov JF, Naber KG, Pilatz A, Wagenlehner FM. Validation of the American English Acute Cystitis Symptom Score. Antibiotics (Basel). 2020 Dec 19;9(12):929. DOI: 10.3390/antibiotics9120929

FIRST VISIT – Part A (diagnostic part) Time: : Date of evaluation: / / (mm/dd/yyyy)

		**Please indicate whether you have had the following symptoms during the past 24 hours, and how severe they were *(Please mark only one answer for each symptom)*:**	**0**	**1**	**2**	**3**
**Typical symptoms**	**1**	Frequent urination of small amounts of urine *(going to the toilet very often)*	☐ None *up to 4 times per day*	☐ Yes, mild *5–6 times/day*	☐ Yes, moderate *7–8 times/day*	☐ Yes, severe *9–10 or more times/day*
	**2**	Urgent urination *(a sudden and uncontrollable urge to urinate)*	☐ None	☐ Yes, mild	☐ Yes, moderate	☐ Yes, severe
	**3**	Feeling burning pain when urinating	☐ None	☐ Yes, mild	☐ Yes, moderate	☐ Yes, severe
	**4**	Feeling incomplete bladder emptying (*Still feel like you need to urinate after urination*)	☐ None	☐ Yes, mild	☐ Yes, moderate	☐ Yes, severe
	**5**	Feeling pain not associated with urination in the lower abdomen *(below the belly button)*	☐ None	☐ Yes, mild	☐ Yes, moderate	☐ Yes, severe
	**6**	Blood seen in urine *(without menses)*	☐ None	☐ Yes, mild	☐ Yes, moderate	☐ Yes, severe
				**Sum of "Typical" scores=**		**points**
**Differential**	**7**	Flank pain *(pain in one or both sides of the lower back)*	☐ None	☐ Yes, mild	☐ Yes, moderate	☐ Yes, severe
	**8**	Abnormal vaginal discharge *(abnormal amount, color and/or odor)*	☐ None	☐ Yes, mild	☐ Yes, moderate	☐ Yes, severe
	**9**	Discharge from the urethra (*urinary opening*) without urination	☐ None	☐ Yes, mild	☐ Yes, moderate	☐ Yes, severe
	**10**	Feeling high body temperature/fever<br>Temperature measured ☐ No ☐ Yes	☐ None (≤99.5˚F)	☐ Yes, mild (99.6˚F–100.2˚F)	☐ Yes, moderate (100.3°F–102.0°F)	☐ Yes, severe (≥102.1 ˚F)
				**Sum of "Differential" scores=**		**points**

**Quality of life**	**11**	**Please rate how much discomfort you have experienced because of these symptoms in the past 24 hours *(Please mark only one answer)*:**<br>☐ 0 No discomfort *(No symptoms at all. I feel as good as usual)*<br>☐ 1 Mild discomfort *(I feel a little worse than usual)*<br>☐ 2 Moderate discomfort *(I feel much worse than usual)*<br>☐ 3 Severe discomfort *(I feel terrible)*
	**12**	**Please indicate how these symptoms have interfered with your everyday activities/work in the past 24 hours *(Please mark only one answer)*:**<br>☐ 0 Did not interfere at all *(Working as usual on a working day)*<br>☐ 1 Mildly interfered *(Due to the symptoms, I work slightly less)*<br>☐ 2 Moderately interfered *(Daily work requires effort)*<br>☐ 3 Severely interfered *(I almost cannot work)*
	**13**	**Please indicate how these symptoms have interfered with your social activities *(visiting people, meeting with friends, etc.)* in the past 24 hours *(Please mark only one answer):***<br>☐ 0 Did not interfere at all *(My social activities did not change in any way, I live as usual)*<br>☐ 1 Mildly interfered (*Insignificant decrease in activities)*<br>☐ 2 Moderately interfered *(Significant decrease. I have to spend more time at home)*<br>☐ 3 Severely interfered *(It's terrible. I barely left the house)*
		**Sum of "QoL" scores= points**

		**14 Please indicate whether you have the following at the time of completion of this questionnaire:**		
**Additional**	**14**	Menstruation (*menses*)?	☐ No	☐ Yes
		Premenstrual syndrome *(PMS)*?	☐ No	☐ Yes
		Signs of menopausal syndrome *(e.g. hot flashes)*?	☐ No	☐ Yes
		Pregnancy?	☐ No	☐ Yes
		Known (diagnosed) diabetes mellitus *(high sugar)*?	☐ No	☐ Yes

FOLLOW-UP VISIT – Part B (patient-reported outcome)

Time: : Date of evaluation: / /

Please indicate if you experienced any changes in your symptoms since the first time you completed this

Dynamics

- ☐ 0 Yes, I feel back to normal *(All symptoms are completely gone)*
- ☐ 1 Yes, I feel much better *(Most of the symptoms are gone)*
- ☐ 2 Yes, I feel somewhat better *(Only some symptoms are gone)*
- ☐ 3 No, there are barely any changes *(I still have about the same symptoms)*
- ☐ 4 Yes, I feel worse *(My condition is worse)*

Please indicate whether you have had the following symptoms during the past 24 hours, and how severe they were

		***(Please mark only one answer for each symptom)*:**	0	1	2	3
Typical symptoms	1	Frequent urination of small amounts of urine *(going to the toilet very often)*	☐ None *up to 4 times per day*	☐ Yes, mild *5–6 times/day*	☐ Yes, moderate *7–8 times/day*	☐ Yes, severe *9–10 or more times/day*
	2	Urgent urination *(a sudden and uncontrollable urge to urinate)*	☐ None	☐ Yes, mild	☐ Yes, moderate	☐ Yes, severe
	3	Feeling burning pain when urinating	☐ None	☐ Yes, mild	☐ Yes, moderate	☐ Yes, severe
	4	Feeling incomplete bladder emptying *(Still feel like you need to urinate again after urination)*	☐ None	☐ Yes, mild	☐ Yes, moderate	☐ Yes, severe
	5	Feeling pain not associated with urination in the lower abdomen *(below the belly button)*	☐ None	☐ Yes, mild	☐ Yes, moderate	☐ Yes, severe
	6	Blood seen in urine *(without menses)*	☐ None	☐ Yes, mild	☐ Yes, moderate	☐ Yes, severe
				**Sum of "Typical" scores=**		**points**
Differential	7	Flank pain *(pain in one or both sides of the lower back)*	☐ None	☐ Yes, mild	☐ Yes, moderate	☐ Yes, severe
	8	Abnormal vaginal discharge *(abnormal amount, color and/or odor)*	☐ None	☐ Yes, mild	☐ Yes, moderate	☐ Yes, severe
	9	Discharge from the urethra *(urinary opening)* without urination	☐ None	☐ Yes, mild	☐ Yes, moderate	☐ Yes, severe
	10	Feeling high body temperature/fever<br>Temperature measured ☐ No ☐ Yes	☐ None (≤99.5˚F)	☐ Yes, mild (99.6˚F–100.2˚F)	☐ Yes, moderate (100.3˚F–102.0˚F)	☐ Yes, severe (≥102.1 ˚F)
				**Sum of "Differential" scores=**		**points**

Quality of life

11 Please rate how much discomfort you have experienced because of these symptoms in the past 24 hours *(Please mark only one answer)*:

- ☐ 0 No discomfort *(No symptoms at all. I feel as good as usual)*
- ☐ 1 Mild discomfort *(I feel a little worse than usual)*
- ☐ 2 Moderate discomfort *(I feel much worse than usual)*
- ☐ 3 Severe discomfort *(I feel terrible)*

☐

12 Please indicate how these symptoms have interfered with your everyday activities/work in the past 24 hours *(Please mark only one answer)*:

- ☐ 0 Did not interfere at all *(Working as usual on a working day)*
- ☐ 1 Mildly interfered *(Due to the symptoms, I work slightly less)*
- ☐ 2 Moderately interfered *(Daily work requires effort)*
- ☐ 3 Severely interfered *(I almost cannot work)*

13 Please indicate how these symptoms have interfered with your social activities *(visiting people, meeting with friends, etc.)* in the past 24 hours *(Please mark only one answer)*:

- ☐ 0 Did not interfere at all *(My social activities did not change in any way, I live as usual)*
- ☐ 1 Mildly interfered *(Insignificant decrease in activities)*
- ☐ 2 Moderately interfered *(Significant decrease. I have to spend more time at home)*
- ☐ 3 Severely interfered *(It's terrible. I barely left the house)*

Sum of "QoL" scores= points

Additional

14 Please indicate whether you have the following at the time of completion of this questionnaire:

Menstruation *(Menses)*?	☐ No	☐ Yes
Premenstrual syndrome *(PMS)*?	☐ No	☐ Yes
Signs of menopausal syndrome *(e.g. hot flashes)*?	☐ No	☐ Yes
Pregnancy?	☐ No	☐ Yes
Known *(diagnosed)* diabetes mellitus *(high sugar)*?	☐ No	☐ Yes
